# Changes in healthcare utilization and costs associated with sildenafil therapy for pulmonary arterial hypertension: a retrospective cohort study

**DOI:** 10.1186/1471-2466-12-75

**Published:** 2012-12-11

**Authors:** Ariel Berger, John Edelsberg, Simon Teal, Marko A Mychaskiw, Gerry Oster

**Affiliations:** 1 Policy Analysis Inc. (PAI), 4 Davis Court, Brookline, MA, 02445, USA; 2 Pfizer Ltd., Walton Oaks, Dorking Road, Walton-on-the-Hill, Tadworth, Surrey, KT20 7NS, UK; 3 Pfizer Inc., New York, NY, 10017, USA

**Keywords:** Pulmonary arterial hypertension, Primary pulmonary hypertension, Sildenafil, PDE5, Phosphodiesterase type 5, Health expenditure, Utilization

## Abstract

**Background:**

Little is known concerning the degree to which initiation of sildenafil for pulmonary arterial hypertension (PAH) impacts patterns of healthcare utilization and costs.

**Methods:**

Using a large US health insurance claims database, we identified all patients with evidence of PAH (ICD-9-CM diagnosis codes 416.0, 416.8) who received sildenafil between 1/1/2005 and 9/30/2008. Date of the first-noted prescription for sildenafil was designated the “index date,” and claims data were compiled for all study subjects for 6 months prior to their index date (“pretreatment”) and 6 months thereafter (“follow-up”); patients with incomplete data during either of these periods were excluded. Healthcare utilization and costs were then compared between pretreatment and follow-up for all study subjects.

**Results:**

A total of 567 PAH patients were identified who began therapy with sildenafil and met all other study entry criteria. Mean (SD) age was 52 (10) years; 73% were women. Healthcare utilization was largely unchanged between pretreatment and follow-up, the only exceptions being decreases in the mean number of emergency department visits (from 0.7 to 0.5 per patient; p < 0.01) and the percentage of patients hospitalized (from 35% to 29%; p = 0.01). The mean cost of all PAH-related medication was $7139 during pretreatment and $14,095 during follow-up (sildenafil cost during follow-up = $5236); exclusive of PAH-related medications, however, total healthcare costs decreased modestly (from $30,104 to $27,605) (p < 0.01 for all comparisons).

**Conclusions:**

The cost of sildenafil therapy may be partially offset by reductions in other healthcare costs.

## Background

Pulmonary arterial hypertension (PAH) is characterized by a pathological narrowing of the pulmonary arterioles and small arteries, which causes elevated pulmonary vascular resistance and increased pressure in the pulmonary arteries and eventually results in the development of right ventricular failure and death [1,2]. Dyspnea, fatigue, chest pain, and syncope are the principal presenting symptoms of PAH [3]. The disease is one form of pulmonary hypertension (broadly defined as increased pressure in the pulmonary arteries, capillaries, or veins). In recent classification schemes for pulmonary hypertension (Dana Point classification [4], guidelines of the European Society of Cardiology and European Respiratory Society [1]), PAH constitutes Group 1 and includes both idiopathic PAH and PAH associated with other specific diseases (Group 2 includes patients with pulmonary hypertension primarily due to left heart disease, Group 3 comprises those with pulmonary hypertension due to chronic pulmonary disease, Group 4 includes cases of chronic thromboembolic pulmonary hypertension, and Group 5 includes miscellaneous types of pulmonary hypertension that do not fit into the other four categories). In epidemiologic studies, the most common types of PAH (in order of decreasing frequency) are: (1) idiopathic; (2) PAH associated with connective tissue disease; and (3) PAH associated with congenital systemic-to-pulmonary shunts in the heart [5-9]. Worldwide, it is estimated that 130,000 to 260,000 persons have PAH [10]. Mean age at diagnosis is >50 years, and the disease is more common among women than men. Most patients present with moderate-to-severe disease and prognosis is poor; 5-year survival in the absence of treatment is only about 50% [9].

The goal of therapy in PAH is to control symptoms of the disease and hopefully slow its progression. Conventional therapy has included the management of underlying or contributing factors, avoidance of pregnancy, early treatment of respiratory tract infections, and immunization against pneumococcal disease and influenza [11]. Calcium channel blockers at high doses also have been an important component of conventional therapy in the small percentage of PAH patients who respond to such therapy.

In recent years, a number of targeted pharmacotherapies have been introduced for the treatment of PAH [12]. There are three main classes of such agents, which act on three main intracellular pathways: (1) prostaglandin/prostacyclin analogues (e.g., intravenous epoprostenol, nebulized or intravenous iloprost); (2) endothelin receptor antagonists (e.g., oral bosentan); and (3) phosphodiesterase-type 5 (PDE-5) inhibitors (e.g., oral sildenafil). These targeted therapies have been shown to improve exercise capacity, hemodynamics, symptoms, and health-related quality of life [13].

Sildenafil (Revatio®) is a PDE-5 inhibitor that was approved for the treatment of PAH (to improve exercise capacity) in 2005 in the US and the European Union, and then in 2009, to delay clinical worsening (US only) [14]. While the efficacy and safety of sildenafil are well established, comparatively little is known about the effects of such therapy on healthcare utilization and costs in “real-world” settings. Our study examined this issue using health insurance claims data.

## Methods

### Data source

Data were obtained from the Medstat MarketScan Commercial Claims and Encounters Database. The database is comprised of facility, professional-service, and retail (i.e., outpatient) pharmacy claims from a variety of private insurers, providing healthcare coverage to approximately 15 million persons annually throughout the US. All patient identifiers in the database have been fully encrypted, and the database is fully compliant with the Health Insurance Portability and Accountability Act of 1996. As no patient or provider contact was made, and patient information was de-identified; institutional review board (IRB) approval was not required.

Information available for each facility and professional-service claim includes date and place of service, diagnoses (in International Classification of Diseases, 9^th^ revision, Clinical Modification [ICD-9-CM] format), procedures (in ICD-9^-^CM [selected plans only], Current Procedural Terminology 4^th^ Edition, and Healthcare Common Procedure Coding System formats), provider specialty, and charged and paid amounts. Data available for each retail pharmacy claim include the drug dispensed (in National Drug Code format), the dispensing date, and the quantity dispensed and number of days of therapy supplied (selected plans only). All claims include a charged amount; the database also provides paid (i.e., reimbursed, including patient deductible, copayment, and/or coinsurance) amounts.

Selected demographic and eligibility information is also available, including age, gender, geographic region, coverage type, and the dates of insurance coverage. All patient-level data can be arrayed in chronologic order to provide a detailed, longitudinal profile of all medical and pharmacy services used by each plan member. The database for this study encompassed the period, January 1, 2005 through September 30, 2008 (“study period”).

### Study sample

The source population for our study consisted of all persons with any inpatient claims, or two or more outpatient claims at least 30 days apart, with a diagnosis of pulmonary hypertension (ICD-9-CM diagnosis codes 416.0 [primary pulmonary hypertension] or 416.8 [secondary pulmonary hypertension]) between January 1, 2005 and September 30, 2008. We included patients with either of these diagnosis codes (i.e., primary or secondary pulmonary hypertension) to ensure complete capture of all those with PAH, since the ICD-9-CM coding system does not coincide with contemporary classification schemes for pulmonary hypertension. Among these patients, we then identified those with one or more pharmacy claims for Revatio, the commercial name of sildenafil that is indicated for the treatment of PAH (sildenafil is also sold under the brand name of Viagra® for erectile dysfunction; the dosages of Revatio and Viagra differ, however, as do the number of pills supplied per respective prescription). The date of each patient’s first-noted claim for Revatio was designated his or her “index date”, and claims data were compiled for all study subjects for 6 months prior to their index date (“pretreatment”) and 6 months thereafter (“follow-up”). (Revatio is indicated only for the treatment of PAH [14], we therefore assumed that it was initiated only for this disease among patients in our study sample, and consequently did not require that the diagnostic evidence of PAH occur prior to the index date.) Patients were excluded from the study sample if they: (1) had incomplete data during pretreatment or follow-up; (2) received Viagra during pretreatment; (3) were aged <18 years as of their index date; or (4) were aged ≥65 years as of their index date if they were not enrolled in a Medicare risk-sharing (i.e., capitated) plan. While we excluded patients who received Viagra in the pretreatment period, receipt of Viagra during follow-up was not an exclusion criterion (i.e., study subjects were required to have initiated therapy with Revatio, but could have switched to Viagra for reasons related to dosage or cost).

### Measures and analyses

We examined selected demographic and clinical characteristics of study subjects, including the number with various comorbidities (Table 1), based on information during the 6-month pretreatment period. Patients were assumed to have a given condition if they had evidence during pretreatment of either one or more hospitalizations, or two or more outpatient claims at least 30 days apart, with a corresponding diagnosis code and/or prescription. The Charlson comorbidity index also was calculated [15].

**Table 1:** Comorbidities of interest

**Comorbidity**	**Definition**
Connective tissue diseases	ICD-9-CM diagnoses 710.0, 710.1, 714.X, 710.3, 710.4
Congenital heart diseases	ICD-9-CM diagnoses 745.3, 745.4, 745.5, 745.6, 747.0
HIV/AIDS	ICD-9-CM diagnoses 042, 079.53, V08
Depressive disorders	ICD-9-CM diagnoses 311, 296.2X, 296.3X, 296.5X, 296.82, 300.4, 298.0, 309.0, 309.28, 309.1
Anxiety disorders	ICD-9-CM diagnoses 300.XX, 301.XX, 309.21
Sleep disorders	ICD-9-CM diagnoses 780.57, 780.50, 780.51, 780.53, 307.4X, 780.5X, V69.4
Lung disease	
Asthma	ICD-9-CM diagnoses 493.XX
Chronic obstructive pulmonary disease	ICD-9-CM diagnoses 491.XX, 492.XX, 496.XX
Pulmonary fibrosis	ICD-9-CM diagnoses 515, 516.3
Cerebrovascular disease	ICD-9-CM diagnoses 430-438.XX
Coronary heart disease	ICD-9-CM diagnoses 410-414.XX
Atrial fibrillation	ICD-9-CM diagnoses 427.3
Congestive heart failure	ICD-9-CM diagnoses 428.XX
Peripheral vascular disease	ICD-9-CM diagnoses 440.2X, 440.3X, 443.9X, 444.22
Hemtaological conditions	ICD-9-CM diagnoses 282.6, 282.49, 289.6, 205.1X
Liver disease	ICD-9-CM diagnoses 572.3, 006.3, 070.22, 070.32, 070.33, 070.44, 070.54, 456.0-456.21, 570-572.29, 572.4- 573.9, 996.82, V42.7
Renal disease	ICD-9-CM diagnoses 293.9, 294.8, 276.0-276.9, 458.21, 567.XX, 584.XX-586.XX, 792.5, 996.1, 996.62, 996.56, 996.68, 996.73, 999.2, 999.3, 999.9, V45.1, V56.0, V56.1, V56.2, V56.3X, V56.8, E87.02, E87.91; ICD-9-CM procedures 39.42, 39.43, 39.93, 39.94, 39.9
Diabetes	ICD-9-CM diagnoses 250.XX; receipt of alpha-glucosidase inhibitors, insulin, metformin, nonsulfonylurea insulin secretagogues, sulfonylurea, or thiazolidinedione
Neoplasms	ICD-9-CM diagnosis 140.XX-209.XX, 230.XX-238.XX

HIV/AIDS: Human immunodeficiency virus/Acquired Immune Deficiency Syndrome.

Levels of healthcare utilization and cost were examined during pretreatment and follow-up, including services and medications related to the treatment of PAH. PAH-related services were identified based on claims for medical treatment with an ICD-9-CM diagnosis code for PAH. PAH-related medications were assumed to consist of PDE-5 inhibitors (including both Revatio and Viagra), prostaglandin/prostacyclin analogues, endothelin receptor antagonists, nitric oxide/nitric oxide donors, calcium channel blockers, oral anticoagulants/antiplatelets, diuretics, oxygen, and cardiac glycosides. (While many of these medications are prescribed for conditions other than PAH [e.g., calcium channel blockers for hypertension and coronary artery disease], we designated them as “PAH-related” given their potential for use in the treatment of PAH).

The statistical significance of differences in continuous measures was assessed using paired t-tests and Wilcoxon signed-rank tests for measures that were normally and non-normally distributed, respectively; for categorical data, McNemar’s and Bowker’s tests were used, as appropriate. All analyses were conducted using PC-SAS® v.8.4.

## Results

We identified a total of 567 patients with evidence of PAH who began therapy with sildenafil and satisfied all other entry criteria (Table 2). Eighty-six percent of patients in the study sample had encounters with ICD-9-CM diagnoses of both primary pulmonary hypertension (416.0) and secondary pulmonary hypertension (416.8) during the study period; the remaining 14% had claims only for primary (4.3%) or secondary (9.7%) pulmonary hypertension. Mean (standard deviation) age of study subjects was 52.3 (9.8) years; 72.7% were women (Table 3). The percentage of study subjects with selected comorbidities was as follows: connective tissue disease, 18.3%; congenital heart disease; 4.1%; chronic obstructive pulmonary disease, 19.0%; pulmonary fibrosis, 12.2%; and congestive heart failure, 23.6%.

**Table 2:** Sample selection

**Criteria**	**Number of patients**
Total number of patients with ≥1 inpatient claims, or ≥2 outpatient claims at least 30 days apart, with diagnosis of PAH during study period* and	22101
One or more pharmacy claims for Revatio during study period and	1116
≥6 months enrollment prior to index date** and	899
Eligible for medical and pharmacy benefits for duration of study period and	899
Were aged ≥18 years as of index date and	856
Were aged <65 years as of index date or	856
Were aged ≥65 years as of index date and enrolled in Medicare	856
Had total costs ≥ $0 and	855
Had no receipt of Viagra during pre-index period and	793
Had ≥6 months enrollment following index date**	567

*Spanning January 1, 2005 to end of database.

**Defined as the date of the first-noted claim for Revatio.

**Table 3:** Demographic and clinical characteristics of study subjects (N = 567*)

**Characteristic**	**Value**
Mean (SD) age, y	52.3 (9.8)
Number (%) women	412 (72.7)
Comorbidities (n [%])	
Connective tissue diseases	104 (18.3)
Congenital heart diseases	23 (4.1)
HIV/AIDS	1 (0.2)
Depressive disorders	29 (5.1)
Anxiety disorders	8 (1.4)
Sleep disorders	83 (14.6)
Lung disease	
Asthma	33 (5.8)
Chronic obstructive pulmonary disease	108 (19.0)
Pulmonary fibrosis	69 (12.2)
Any of above	172 (30.3)
Cerebrovascular disease	10 (1.8)
Coronary heart disease	68 (12.0)
Atrial fibrillation	46 (8.1)
Congestive heart failure	134 (23.6)
Peripheral vascular disease	9 (1.6)
Hematological conditions	8 (1.4)
Liver disease	44 (7.8)
Renal disease	72 (12.7)
Diabetes	91 (16.0)
Neoplasms	38 (6.7)
Mean (SD) Charlson comorbidity index	1.0 (1.1)

PAH: Pulmonary arterial hypertension; HMO: Health maintenance organization; PPO: Preferred provider; HIV/AIDS: Human immunodeficiency virus/Acquired Immune Deficiency Syndrome.

**Figure 1:**
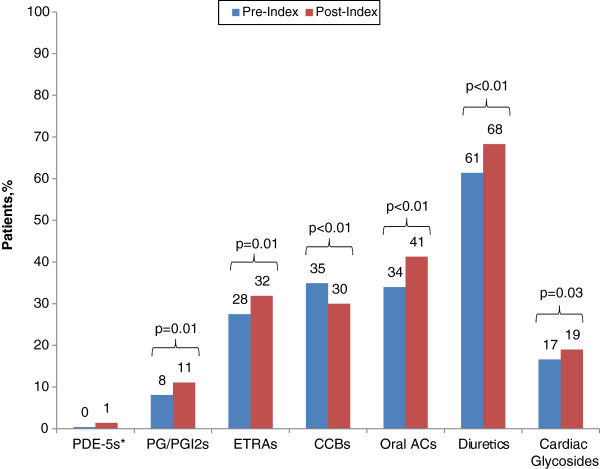
**Use of PAH-related pharmacotherapy during pre- and post-index periods.** *Excluding sildenafil. PDE-5: Phosphodiesterase type-5 inhibitor; PG/PGI2: Prostaglandin/prostacyclin analogues; ETRA: Endothelin-receptor antagonist; CCB: Calcium-channel blocker; AC: Anticoagulant.

Use of most PAH-related medications increased between pretreatment and follow-up, including prostaglandin/prostacyclin analogues (8.1% of patients received these agents during pretreatment vs 11.1% during follow-up [p = 0.01]), endothelin receptor antagonists (27.5% to 31.9% [p = 0.01]), oral anticoagulants (34.0% to 41.3% [p < 0.01]), diuretics (61.4% to 68.3% [p < 0.01]), and cardiac glycosides (16.6% to 19.0% [p = 0.03]) (Figure 1). Use of calcium channel blockers declined (from 34.9% to 30.0%) (p < 0.01). There were few statistically significant differences in healthcare utilization between pretreatment and follow-up, the only exceptions being declines in the mean number of emergency department (ED) visits per patient (from 0.7 to 0.5 per patient; p < 0.01) and the percentage of patients hospitalized (from 35.1% to 28.9%; p = 0.01) (Table 4).

**Table 4:** Use of healthcare services during pre- and post-index periods

**Service**	**Pre-index**	**Post-index**	***P*-value**
Outpatient services			
Physician office visits			
Number (%) with ≥1 visits	561 (98.9)	559 (98.6)	0.48
Number of visits			
Mean (95% CI)	11.7 (11.1, 12.4)	12.1 (11.3, 12.9)	0.60
Median (IQR)	10 (6, 15)	10 (6, 16)	
Other outpatient office visits			
Number (%) with ≥1 visits	550 (97.0)	540 (95.2)	0.08
Number of visits			
Mean (95% CI)	10.1 (9.4, 10.9)	10.3 (9.5, 11.1)	0.52
Median (IQR)	8 (5, 13)	8 (4, 14)	
ED visits			
Number (%) with ≥1 visits	194 (34.2)	140 (24.7)	<0.01
Number of visits			
Mean (95% CI)	0.7 (0.6, 0.8)	0.5 (0.4, 0.7)	<0.01
Median (IQR)	0 (0, 1)	0 (0, 0)	
Hospitalizations			
Number (%) with ≥1 hospitalizations	199 (35.1)	164 (28.9)	0.01
Number of hospitalizations			
Mean (95% CI)	0.5 (0.4, 0.6)	0.5 (0.4, 0.5)	0.18
Median (IQR)	0 (0, 1)	0 (0, 1)	
Length of stay			
All patients			
Mean (95% CI)	4.3 (3.5, 5.1)	3.9 (3.0, 4.7)	0.06
Median (IQR)	0 (0, 5)	0 (0, 3)	
Patients with ≥1 hospitalizations			
Mean (95% CI)	8.8 (7.3, 10.3)	7.8 (6.2, 9.5)	0.06
Median (IQR)	5 (0, 11)	3 (0, 9)	

The mean cost of PAH-related medication increased from $7139 during pretreatment to $14,095 during follow-up (p < 0.01). The cost of PDE-5 inhibitors was $5236 during follow-up, almost all of it attributable to Revatio (5 patients had evidence of receipt of Viagra; 3 patients, tadalafil). Mean total healthcare costs increased from $37,243 during pretreatment to $41,700 during follow-up; exclusive of the cost of PAH-related pharmacotherapy, mean total healthcare costs declined from $30,104 to $27,605 (both p < 0.01) (Table 5). Significant reductions were noted in the mean cost of physician office visits (from $2088 to $1935), other outpatient visits (from $6226 to $5490), and emergency department visits (from $355 to $310) (all p < 0.01). There was a nominal, albeit not statistically significant, decline in the mean cost of hospitalization—from $13,743 during pretreatment to $11,602 during follow-up (p = 0.18), most likely reflective of the aforementioned decline in the percentage of patients admitted to hospital.

**Table 5:** Mean total healthcare costs during pre- and post-index periods*

		**22,101**	
	**Pre-index**	**Post-index**	***P*-value**
Pharmacotherapy			
PAH-related			
Phosphodiesterase type-5			
inhibitors	2 (0, 4)	5,236 (4963, 5510)	<0.01
Prostaglandin/prostacyclin			
analogues	1,569 (934, 2204)	2,271 (1574, 2969)	<0.01
Endothelin receptor antagonists	4,686 (4009, 5363)	5,613 (4867, 6359)	<0.01
Calcium channel blockers	79 (66, 92)	71 (59, 83)	0.14
Oral anticoagulants	31 (26, 36)	41 (35, 47)	<0.01
Diuretics	61 (52, 71)	75 (64, 87)	<0.01
Oxygen	708 (601, 816)	784 (684, 883)	<0.01
Cardiac glycosides	4 (3, 5)	5 (4, 6)	<0.01
Any of above	7,139 (6122, 8156)	14,095 (12982, 15209)	<0.01
All other	2,714 (2041, 3386)	2,876 (2323, 3428)	0.10
Total pharmacotherapy	9,853 (8614, 11091)	16,971 (15758, 18184)	<0.01
Outpatient services			
Physician office visits	2,088 (1807, 2368)	1,935 (1603, 2267)	<0.01
Other outpatient office visits	6,226 (5060, 7391)	5,490 (4245, 6736)	<0.01
ED visits	355 (270, 440)	310 (209, 411)	<0.01
Total outpatient services	8,668 (7439, 9898)	7,735 (6401, 9070)	<0.01
Hospitalizations	13,743 (9745, 17741)	11,602 (8443, 14762)	0.18
All other	4,979 (3627, 6332)	5,391 (3920, 6863)	0.96
Total			
Exclusive of PAH-related			
medications	30,104 (25422, 34787)	27,605 (23536, 31674)	<0.01
Inclusive of PAH-related			
medications	37,243 (32467, 42020)	41,700 (37470, 45931)	<0.01

*All values are mean (95% CI) total healthcare costs, $.

## Discussion

Mean total healthcare costs during the six-month period following initiation of sildenafil therapy were higher than they were in the period immediately preceding the start of such therapy, due largely to increases in the cost of PAH-related pharmacotherapy. Exclusive of the cost of pharmacotherapy, mean total healthcare costs declined by about $2500, primarily as a result of significant reductions in the cost of physician office visits (−$153), other outpatient visits (−$736), and emergency department visits (−$45).

Since the database that we used does not contain any information on clinical effectiveness *per se* (e.g., six-minute walk test, cardiopulmonary hemodynamics), our study provides no direct evidence thereof for sildenafil. We believe, however, that our study nonetheless provides some indirect evidence of its effectiveness, if one assumes that costs of care are a reasonably accurate mirror of disease progression and severity. Our finding that the cost of PAH-related inpatient and outpatient care (i.e., exclusive of the cost of PAH-related pharmacotherapy) declined would appear to be consistent with the hypothesis that the efficacy of sildenafil is manifested in clinical practice via reductions in the costs of PAH-related services [13,16-24]. Our finding that initiation of sildenafil therapy appears to be coupled in many patients with increased use of other PAH-related medications is not surprising in a progressive disease such as PAH.

Of course, there are other possible explanations for the reductions in the cost of PAH-related care that we observed. For one, the use of other PAH-related medications, such as prostaglandin/prostacyclin analogues (e.g., iloprost) and endothelin-receptor antagonists (e.g., bosentan), also increased significantly during follow-up. The decline in healthcare costs that we observed may simply be a result of better tailoring of medication regimens to patients’ needs and not to the use of sildenafil alone. Cost reductions also could reflect regression to the mean, if there is lability in signs and symptoms and clinicians are more likely to modify medication regimens when patients are doing poorer clinically (e.g., experiencing exacerbation). Given the scant clinical data available to us, the precise reason(s) for observed reductions in healthcare costs following initiation of sildenafil therapy must remain conjectural.

Certain limitations of our study warrant mention. We could not definitively identify patients with PAH because ICD-9-CM coding for pulmonary hypertension predates current classification schemes. We included patients who received either of the two principal diagnosis codes for pulmonary hypertension (416.0, 416.8) to ensure complete capture of all patients with PAH. Although 90% of study subjects had at least one claim for primary pulmonary hypertension (presumably, idiopathic or heritable PAH), most (86%) also had claims for other forms of pulmonary hypertension, which could include PAH associated with other disorders (e.g., PAH due to connective tissue disease), as well as other, non-PAH, pulmonary hypertension . The relatively high prevalence of both congestive heart failure (CHF) (a common underlying disease in Group 2 pulmonary hypertension) and chronic obstructive pulmonary disease (COPD) (a common underlying disease in Group 3 pulmonary hypertension) in our study population (24% and 19%, respectively) further suggests that some degree of misclassification may have occurred. Although it is possible to have both PAH and COPD and/or CHF (right-heart failure develops late in the course of PAH), the prevalence of both PAH and CHF or COPD is probably small, which raises the possibility that a proportion of these patients in our sample had Group 2 and Group 3 pulmonary hypertension, respectively. We note, however, that even if there were no overlap between patients with CHF and those with COPD, and all patients with CHF or COPD had secondary pulmonary hypertension, this would account for only about one-half of all patients with ICD-9-CM diagnosis codes for both primary and secondary pulmonary hypertension.

Furthermore, we believe that most study subjects with ICD-9-CM diagnosis codes for primary and secondary pulmonary hypertension had PAH. All study subjects had to have evidence of receipt of sildenafil, which has been approved for the treatment of PAH but not other classes of pulmonary hypertension. We acknowledge that in clinical practice, physicians may prescribe drugs approved exclusively for PAH for patients with other forms of pulmonary hypertension, but note that the inclusion of such patients in our study sample should have imparted a conservative bias to our findings because the costs of sildenafil therapy would have been incurred without the benefits in terms of decreased utilization and cost of healthcare services. The degree to which misclassification actually occurred in our study must remain conjectural.

Finally, we note that we did not include a concurrent control group as part of our study, and that there are well-established threats to validity associated with these types of research designs. While we were mindful of the problems posed by the use of a study design that did not utilize a concurrent control, we were similarly concerned about the comparability of any population of control subjects that we might have designated. Specifically, it would have required that we identify a group of patients beginning therapy with something other than a PDE-5 inhibitor, and then compare changes in healthcare costs (i.e., pre-treatment versus post-initiation) between these patients and those beginning therapy with Revatio. Underlying differences in disease severity and other potentially important clinical parameters, however, could introduce confounding and bias into such a comparison if not adequately controlled for in the analyses. In the end, we elected not to include concurrent controls in our study due these problems. This significant limitation of our study design should be borne in mind, however, and further study would be needed to ascertain more rigorously the true effects of Revatio therapy for PAH.

## Conclusions

In conclusion, while total healthcare costs increased among PAH patients initiating therapy with sildenafil, the cost of such therapy may be partially offset by reductions in other healthcare costs—specifically, those for outpatient and inpatient services.

## Competing interests

All authors reviewed and contributed to the study research plan, interpretation of the data, and the study manuscript. Data management, processing, and analyses were conducted by AB, JE, and GO. All authors read and approved the final manuscript. AB and GO take responsibility for the integrity of the work as a whole, from inception to published article.

The study was sponsored by Pfizer Inc. AB, JE, and GO are employees of Policy Analysis Inc. who were paid consultants to Pfizer Inc. in connection with the development of this manuscript. ST and MM are employees of Pfizer, Inc.

## Authors’ contributions

All authors (AB, JE, ST, MM and GO) made substantial contributions to study conception and design, interpretation of data, were involved in manuscript preparation and review, and have given final approval of the version to be published. AB, JE, and GO undertook data analyses.

## Financial support

The study was sponsored by Pfizer Inc. AB, JE, and GO are employees of Policy Analysis Inc. who were paid consultants to Pfizer Inc. in connection with the development of this manuscript. ST and MM are employees of Pfizer, Inc.

## Pre-publication history

The pre-publication history for this paper can be accessed here:

http://www.biomedcentral.com/1471-2466/12/75/prepub
